# Laparoscopic myomectomy of a subserous pedunculated fibroid at 14 weeks of pregnancy: a case report

**DOI:** 10.1186/1752-1947-5-545

**Published:** 2011-11-05

**Authors:** Mario Ardovino, Italo Ardovino, Maria Antonietta Castaldi, Antonietta Monteverde, Nicola Colacurci, Luigi Cobellis

**Affiliations:** 1 Department of Gynaecology, Obstetrics and Reproductive Science, Second University of Studies of Naples, Largo Madonna delle Grazie 1, 80138, Naples, Italy; 2 Operative Unit of Obstetrics and Gynecology, A.O.R.N. S.G. Moscati, Avellino, Italy

## Abstract

**Introduction:**

Uterine leiomyomas are seen in 1.6% to 4% of pregnancies. With the increasing age of obstetric patients, more cases are being encountered during pregnancy.

**Case presentation:**

We report the case of a 31-year-old Caucasian woman with acute recurrent abdominal pain due to a subserous fundic myoma, measuring 48 × 52 × 63 mm, with an implantation base of 22 × 18 mm, which was successfully treated by laparoscopy at 14 weeks of pregnancy. At a gestational age of week 40, the patient spontaneously delivered a healthy 3216 g girl baby.

**Conclusion:**

As far as we know, this is the first reported case of laparoscopic myomectomy this early during a pregnancy. Our experience together with an analysis of cases reported in the literature suggests that myomectomy during pregnancy may be considered safe, but only in the hands of experienced laparoscopic surgeons. There are a few reports in the literature about laparoscopic myomectomy during the first half of pregnancy that demonstrate its feasibility in selected cases. Some technical tools could improve the procedure with a minimum of risk for the ongoing pregnancy.

## Introduction

Uterine leiomyomas are seen in 1.6% to 4% of pregnancies. With the increasing age of obstetric patients, more cases are being encountered during pregnancy. The effect of leiomyomas on pregnancy depends on their number, size and location. Myomectomy is generally avoided during pregnancy because increased vascularity can lead to hemorrhagic complications that may necessitate hysterectomy, but it is indicated in some situations [1-3]. Indications for myomectomy during pregnancy include severe abdominal pain due to torsion of subserous pedunculated myomas or red degeneration not responding to medical treatment, and an increase in myoma size causing abdominal discomfort [4,5].

There are a few reports in the literature about laparoscopic myomectomy during the first half of pregnancy that demonstrate its feasibility in selected cases [1,6-10]. However, laparoscopic myomectomy can be considered a minimally invasive alternative to the traditional laparotomy during pregnancy, resulting in less postoperative pain and a shorter recovery time [3].

We report a case of acute recurrent abdominal pain due a subserous fundic myoma that was treated by laparoscopy at 14 weeks of pregnancy. Additionally, we examine some technical surgical aspects and devices, and review the international literature.

## Case Presentation

A 31-year-old, Caucasian primigravid woman was referred to our attention at 14 weeks of gestation because of acute recurrent abdominal pain localized in the right periumbilical region and not responsive to analgesic therapy. On admission she was 165 cm height, 65 kg weight with a body mass index (BMI) of 23.5. She had no fever and a normal white blood cell count.

At pelvic examination the uterus appeared bigger than expected for gestational age and irregular. A trans-abdominal sonographic scan showed the presence of an intrauterine singleton pregnancy corresponding to gestation date and a subserous fundic myoma, measuring 48 × 52 × 63 mm, with an implantation base of 22 × 18 mm. No other ultrasonographic sign related to the symptoms such as hemoperitoneum or area of degeneration in the myoma was observed. She received paracetamol and tramadol, without relief. As the acute abdominal pain symptoms suggested a possible torsion of the myoma, laparoscopic surgery was performed under general anaesthesia.

**Figure 1:**
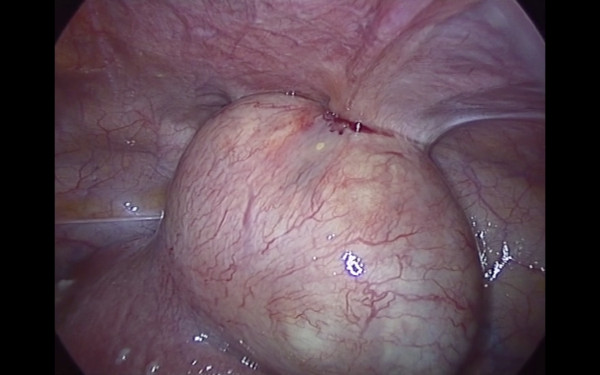
**Subserous myoma, with its implantation base localized in the fundic right lateral region and a consistent adherence to the anterior abdominal wall**.

**Figure 2:**
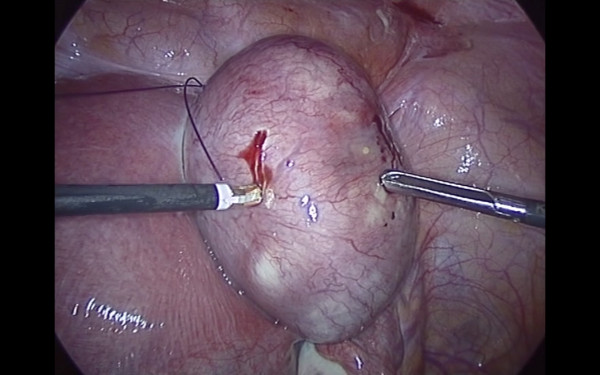
**The ischemic myoma at the beginning of the enucleation by bipolar electrosurgical device (PKS Plasmaspatula, Gyrus Medical Inc, Minneapolis, MN)**.

**Figure 3:**
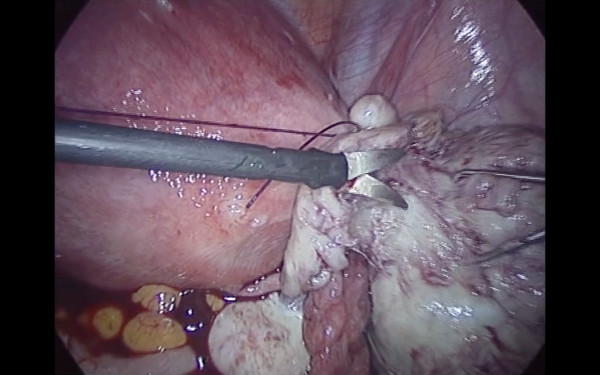
**Myoma enucleation**.

**Figure 4:**
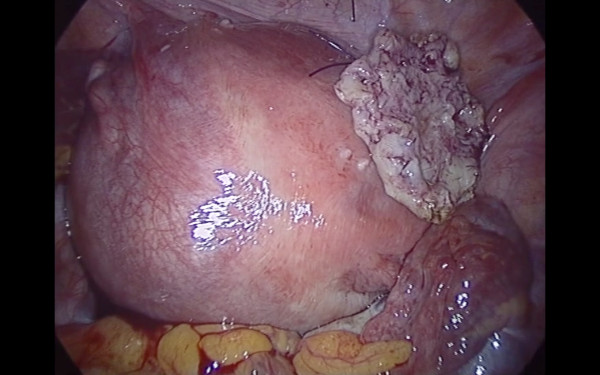
**The uterus at the end of the intervention with the strangled and ischemic minimum residual tissue**.

Pneumoperitoneum was achieved by infra-umbilical Veress needle until an intra-abdominal pressure of 10 mmHg was reached. The first trocar was introduced transumbilically. Intra-abdominal visualization, obtained with a 10 -mm, 0-degree, high definition telescope, showed an enlarged, irregular uterus with a subserous myoma, with an implantation base localized in the fundic right lateral region, with a consistent adherence to the anterior abdominal wall (Figure 1). The adnexa, round ligaments, appendix, and gallbladder were regular. Under laparoscopic vision two 5 mm ancillary trocars were positioned in the right and left pelvic region, 2 cm above and 1 cm medial to the anterior superior iliac spine. The adherence was carefully removed and separated from the myoma with gentle maneuvers. The base of the myoma was identified and strangled with a Monocryl-1, no-needle suture performed with an extracorporeal technique. After waiting for the myoma ischemia, the myoma was enucleated by a bipolar electrosurgical device (PKS Plasmaspatula, Gyrus Medical Inc, Minneapolis, MN), leaving minimal residual tissue (Figure 2, Figure 3 and Figure 4). At this point a 3-mm, 0-degree, high definition telescope was introduced in the left 5-mm trocar and the umbilical trocar was replaced with a Rotocut G1 tissue morcellator (Karl Storz GmbH & Co. KG, Tuttlingen, Germany). The entire myoma was removed from the abdominal cavity by transumbilical morcellation. Blood loss was 30 mL and came only from the myoma. The surgical laparoscopic breaches were closed with biological surgical glue (Glubran 2-synthetic surgical glue, GEM S.r.l., Viareggio, Italy). The operation lasted for 39 minutes. Fetal heart monitoring was regular pre- and post-operatively. No intra- or post-operative complications occurred, and she was discharged after three days.

Definitive pathologic examination confirmed the diagnosis of a myoma weighing 127 g. At gestational age week 40, she spontaneously delivered a healthy baby girl weighing 3216 g.

## Discussion

This is the first reported case of laparoscopic myomectomy during such an early time of pregnancy (14 weeks) that we could find in the literature. Although the myoma was quite big (127 g), its position (fundic right lateral region) let us easily complete the intervention. Although postoperative adhesion development after laparoscopic myomectomy has been described in the literature and in our patient the source of pain was the adhesion, simple adhesiolysis was not contemplated. We decided to perform a complete myomectomy to eliminate the risk of red degeneration and necrosis during the ongoing pregnancy [4,5]. We also decided not to apply an adhesion barrier over the surgical site, since data presented in the literature are still not conclusive, even if they show an encouraging safety profile [11].

Uterine myomas are the most frequent gynecological tumors and they are seen in 1.6% to 4% of pregnancies. Most myomas remain asymptomatic during pregnancy. Routine ultrasonography performed at this time improves the detection of these lesions and the evaluation of any possible complications. Such complications comprise spontaneous abortion, antepartum bleeding, premature rupture of membranes, preterm labor, placenta previa, post-partum bleeding and a high incidence of Caesarean deliveries [2].

Although controversy persists among reports of myomectomy being performed during pregnancy, there are specific clinical conditions that require this surgical procedure. These include severe abdominal pain due to torsion of subserous pedunculated myomas or red degeneration not responding to medical treatment, and an increase in myoma size causing abdominal discomfort [2,4].

Although myomectomy during pregnancy may lead to abortion, fetal acidosis and hemorrhage, we are aware of no available published cases of post-operative abortion or fetal acidosis during laparoscopic myomectomy [2,3]. Hemorrhage is the first complication of laparoscopic myomectomy. Although there is a high incidence of blood loss during myomectomy, there are no reported cases of conversion to open surgery or surgical delivery related to myoma resulting in uncontrolled bleeding during laparoscopy [4,5].

Although a laparoscopic approach for uterine myomas during pregnancy is rarely described, our experience suggests that uterine myomas can be easily managed laparoscopically by an experienced surgeon in selected cases, depending on size, type and position of the fibroids.

A MEDLINE and EMBASE search revealed six case reports of laparoscopic myomectomy during pregnancy from 1994 to 2011, carried out in the second half of pregnancy between 16 and 25 weeks of gestation (Table 1) [1,6-10]. All the procedures were performed for acute abdominal pain, and in five cases pregnancy ended with spontaneous delivery, while only one case underwent preterm delivery (caesarean section) because septic necrosis of the myometrium occurred [8].

**Table 1:** Case reports of laparoscopic myomectomy performed during pregnancy: overview of the characteristics.

Author	Year	N° of myomas	Gestational week at surgery time	Complications	Parturition week	Delivery modality
Lucas V *et al*.	1994	one	NA	__	NA	NA

Pelosi MA *et al*.	1995	one	16 w	__	39 w	caesarean section

Sentilhes L *et al*.	2003	one	17 w	Septic necrosis of the myometrium	37 w	caesarean section

Melgrati *et al*.	2005	one	24 w	__	39 w	spontaneous

Fanfani *et al*.	2010	one	25 w	__	40 w	spontaneous

Son *et al*.	2011	one	18w		39w	spontaneous

NA: not available.

The main issues regarding laparoscopic myomectomy during pregnancy were found to be the following: entry, pneumoperitoneum, electricity, uterine mobilization and myoma morcellation.

Initial access can be safely accomplished with an open (Hasson), Veress needle or optical trocar technique if the location is adjusted according to fundal height, previous incisions, and experience of the surgeon (Level III). There has been much debate regarding abdominal access in the pregnant patient with preferences toward either an open technique or Veress needle. The concern for using the Veress needle technique has largely been based on concerns about the higher likelihood of injury to the uterus or other intra-abdominal organs as fundal height increases [3]. If the site of initial abdominal access is adjusted according to fundal height and the abdominal wall is elevated during insertion, both the Hasson technique and the Veress needle can be safely and effectively utilized [3].

In all the reported cases the entry was performed by an open technique and the site of entry chosen according to fundal height [1,6-10].

Our case is the first report of an uncomplicated entry performed with the classic Veress needle technique. Our choice was determined by two main factors: the first one is that there was a low risk of uterine damage, since the 14-week uterine fundus raised the hypogastrium, being 4 cm under the umbilical scar. The second reason was the fact that the myoma was situated in the right iliac fossa, thus avoiding any injury during the entry [3].

The second main issue is pneumoperitoneum induction, as the pneumoperitoneum using CO_2 _may irritate the uterine tissues and increase the risk of acidosis to the fetus.

The potential effects of CO_2 _insufflation on the pregnant patient and her fetus have led to apprehension over its use. The pulmonary effects of pneumoperitoneum in the pregnant patient and the potential risk for acidosis to the fetus have caused concern and have led some investigators to develop alternative approaches of gasless laparoscopy [6] but in only one case was a gasless surgery performed [6]. In our case the insufflation pressure was set at 10 mmHg from the beginning of the intervention. In our 20 years of laparoscopic experience this reduces postoperative pain, and in our case this method lowered the potential effects of CO_2 _insufflation on the patient and her fetus, with a decreased acidosis and abortion risk [3]. Fetal acidosis and associated fetal instability in CO_2 _pneumoperitoneum have been documented in animal studies, although no long-term effects from these changes have been identified [3,12]. Fetal acidosis with insufflation has not been documented in the human fetus, but concerns over potential detrimental effects of acidosis have led to the recommendation of maternal CO_2 _monitoring [3,12]. Initially, there was debate over maternal blood gas monitoring of arterial carbon dioxide (PaCO_2_) versus end-tidal carbon dioxide (EtCO_2_) monitoring, but the less invasive capnography has been demonstrated to reflect maternal acid/base status adequately in humans [3,12]. Several large studies have documented the safety and efficacy of EtCO_2 _measurements in pregnant women [3] making routine blood gas monitoring unnecessary [12].

Electricity is needed during a laparoscopic myomectomy in order to gain an optimum hemostasis. In case of myomectomy during pregnancy all the authors agree on the use of bipolar electricity to minimize any possible injury to the fetus [1,6-10]. In our case, after positioning a Monocryl 1 no- needle suture, we enucleated the myoma with the bipolar Plasmaspatula, thus avoiding sparking phenomena associated with monopolar energy sources that may endanger the fetus or displaced bowel [3,10]. This is a bipolar electrosurgical device designed to deliver high current and very low voltage to tissue. Tissue impedance is continuously monitored by the instrument, and energy delivery is adjusted accordingly. This system delivers electrosurgical energy through a series of rapid pulses, thereby allowing the tissue to cool briefly and limiting the heating of adjacent tissue. Protein in the vessel walls is denatured and forms a coagulum, which occludes the lumen [13].

Therefore, the cut of the myoma was at the level of the large base right up the suture. The minimum residual tissue of the myoma became completely ischemic, thus avoiding any problem for the patient. This method of removing the myoma lets the surgeon cut far away from the myometrium, thus avoiding a possible uterine contraction, with consequent high risk for abortion.

Uterine mobilization may represent a challenge for the surgeon, as it is not possible to use a uterine manipulator during pregnancy. The size of the myoma itself could also represent another challenge. The largest myoma removed by laparoscopy weighed 1500 g [7].

Myoma morcellation may lead to severe uterine damage, which could be harmful for the ongoing pregnancy. A transumbilical morcellation is described in only one of the reported cases [7]; indeed, transumbilical morcellation is well described in laparoscopic gynecological surgery. The present morcellation technique, performed by using a 3 mm, 10 degree telescope introduced in the left 5 mm trocar, lets the surgeon take a comfortable position, and, in addition, this method keeps the morcellator blade far away from the gravid uterus. This is a clear advantage in the situation of pregnancy, where it is impossible to manipulate the uterus. Additionally, the telescope introduced in the left 5 mm trocar allows optimal spatial vision.

In the end, the use of a mininvasive technique with two 5 mm ancillary trocars resulted in minimal damage to the abdominal organs and a high aesthetic acceptability. In our recent experience the use of two 5 mm ancillary trocars for laparoscopic myomectomy is a good approach for the surgeon and has a high aesthetic acceptability for the woman. It lets the surgeon work without particular problems and allows the easy use of either extra- or intra-corporeal knots.

## Conclusion

Our experience, together with the analysis of literature, suggests that laparoscopic myomectomy during pregnancy may be considered safe in selected cases, even in an early stage, but only in the hands of experienced laparoscopic surgeons.

Safety and pregnancy risk cannot be concluded on the basis of six case reports, only five of which have pregnancy outcome data. Further investigations are needed to improve and better define safety and feasibility of laparoscopic myomectomy during pregnancy.

## Consent

Written informed consent was obtained from the patient for publication of this case report and any accompanying images. A copy of the written consent is available for review by the Editor-in-Chief of this journal.

## Competing interests

The authors declare that they have no competing interests.

## Authors' contributions

MA contributed to the paper as surgeon, in the conception of the manuscript, data collection and critical discussion. IA contributed to the paper as surgeon, in the conception of the manuscript, in data analysis, interpretation and critical discussion. MAC contributed to the paper in the conception and drafting of the manuscript, in data analysis, interpretation and critical discussion. AM contributed to the paper in the analysis of the manuscript, data interpretation and critical discussion. NC contributed to the paper in the conception of the manuscript, data analysis and critical discussion. LC contributed to the paper as surgeon, in the conception of the manuscript, in data analysis, interpretation and critical discussion. All authors read and approved the final manuscript.
